# Laparoscopic resection of a rare gastrointestinal stromal tumor in children

**DOI:** 10.1186/s40064-015-0850-9

**Published:** 2015-02-10

**Authors:** Mario Lima, Tommaso Gargano, Giovanni Ruggeri, Andrea Pession, Arianna Mariotto, Michela Maffi

**Affiliations:** Pediatric Surgery, Policlinico S. Orsola, via Massarenti 11, 40138 Bologna, Italy; Pediatrics, Policlinico S. Orsola, via Massarenti 11, 40138 Bologna, Italy

**Keywords:** Gastrointestinal stromal tumor, Laparoscopy, Children

## Abstract

**Introduction:**

Gastrointestinal stromal tumors (GIST) are uncommon mesenchymal tumors of the gastrointestinal (GI) tract, accounting for 0.1% to 3% of all GI malignancies. Paediatric GIST have an annual incidence of 0.02 per million children, have a female predisposition, are usually located in the stomach (50–60%) and in up to 85% of cases CD117-cKit or PDGFRA mutation is absent, resulting in a decreased efficacy of the target therapy.

**Case description:**

We report the case of an incidentally diagnosed gastric GIST in a 14-year-old boy with multiple malformations. Genetic tests and Kariotype resulted negative.

Recently, an abdominal US visualized an hypoechoic heterogeneous abdominal mass. The common tumor markers resulted negative and the abdominal CT-scan confirmed the presence of a solid round lesion (42×36mm) in contact with the stomach and the pancreas. Laparoscopy allowed the recognition and the removal of the nodular mass at the posterior margin of the stomach. The histopathologic and the molecular biology findings were consistent with a kit-wilde type GIST. Surgical margins were microscopically free of tumor cells.

These results justify the decision not to add other surgical or medical therapy. However, for high risk of recurrence and metastasis, a close follow-up was started.

**Discussion and evaluation:**

GIST are asymptomatic in 10% to 30% of patients or present nonspecific symptoms and signs. These tumors present usually irregular, lobulated and ulcerated. CT-scan of the abdomen and pelvis or magnetic resonance imaging (MRI) are mandatory in the diagnostic work-up. The final diagnosis is based on histology and immunohistochemistry. Surgery is the first-line treatment in patients with localized disease.

**Conclusion:**

Guidelines for the management of pediatric GIST are not presently available for the paucity of reports and data. However it is widely accepted that surgery is the first-line treatment and gross resection with negative microscopic margins can be considered therapeutic and lead to full remission of the pathology. Laparoscopy is a safe surgical approach for the exploration of the abdominal cavity, the evaluation of the disease and the complete removal of the tumor.

## Introduction

Gastrointestinal stromal tumors (GISTs) are uncommon mesenchymal tumors of the gastrointestinal (GI) tract accounting for 0.1% to 3% of all GI malignancies (Gunaydin et al. 2012). In children the true incidence is not available for the rarity of the pathology and the difficulty of the correct diagnosis (Benesch et al. 2009). The most accurate epidemiological data on paediatric GIST (less than 14 years) show an annual incidence of 0.02 per million children (Benesch et al. 2009; Guhyun K et al. 2013), 1.4% of all cases (Park et al. 2006; Jagannathan et al. 2012).

In children, GIST can occur sporadically or concurrently with genetic syndromes, including familial GIST with germline KIT mutation, neurofibromatosis type 1, Carney triad and Carney–Stratakis syndrome (Benesch et al. 2009; Guhyun K et al. 2013; Park et al. 2006; Vaughan et al. 2011).

Generally paediatric GIST has a female predisposition (Gunaydin et al. 2012; Guhyun K et al. 2013; Park et al. 2006; Hayashi et al. 2005; Tran et al. 2013) and occurs in the stomach (Benesch et al. 2009; Guhyun K et al. 2013; Park et al. 2006; Hayashi et al. 2005; Tran et al. 2013) or in the small intestines (Gunaydin et al. 2012; Durham et al. 2004; Hayashi et al. 2005; Tran et al. 2013). In up to 85% of paediatric cases c-kit/CD-117 or PDGFRA (Platelet-Derived Growth Factor Receptor Alpha) mutation is absent and it may result in decreased efficacy in target-based therapy with tyrosine kinase receptor inhibitors (Benesch et al. 2009; Tran et al. 2013).

Guidelines for the management of paediatric GIST are not clear and often derives from the adult literature (Benesch et al. 2009; Hayashi et al. 2005), however it is widely accepted that surgery is the first-line treatment (Gunaydin et al. 2012; Benesch et al. 2009; Hayashi et al. 2005) and gross resection with negative microscopic margins may lead to full remission of the pathology (Gunaydin et al. 2012; Benesch et al. 2009; Durham et al. 2004; Hayashi et al. 2005; Tran et al. 2013). Despite a high rate of metastases, tumor recurrence and limited response to tyrosine kinase inhibitors, paediatric GIST have a relatively benign clinical course (Gunaydin et al. 2012; Benesch et al. 2009; Jagannathan et al. 2012; Tran et al. 2013).

## Case report

We report the case of a 14 year-old boy with an incidental US diagnosis of abdominal solid mass. The patient was born at full-term, with pre-natal diagnosis of a cardiovascular malformation: ventricular septal defect, coarctation of the aorta and bicuspid aortic valve. He was operated at birth and one month later to correct the malformations and the consequently subaortic stenosis. The post-operative course was complicated by sepsis and high pressure hydrocephalus, corrected by a ventriculoperitoneal shunt. The young boy was affected by congenital bilateral sensorineural deafness and at 6 years, a cochlear device was implanted. He suffered of musculo-skeletal malformation at shoulder girdle and growth retardation and of IgM deficiency and IgG progressive decrease. Genetic tests were conducted to evaluate the presence of a syndromic pattern: Kariotype, Connexin 26 mutation (responsible for severe hearing loss), subtelomeric rearrangements, 22q delation, CGH array; that resulted negative.

In the last period the patient was in good general condition, with no symptoms referred. During the auxologic control, the abdominal US visualized an hypoechoic heterogeneous mass (4 cm) in the perihepatic region corrisponding to the 3rd hepatic segment, antero-laterally to the gastric antrum. Lab test revealed a mild neutrophilic leukocytosis and anemia. The common tumor markers: AFP, beta-hCG, CEA, ferritin, LDH, urine HVA/VMA resulted negative.

**Figure 1:**
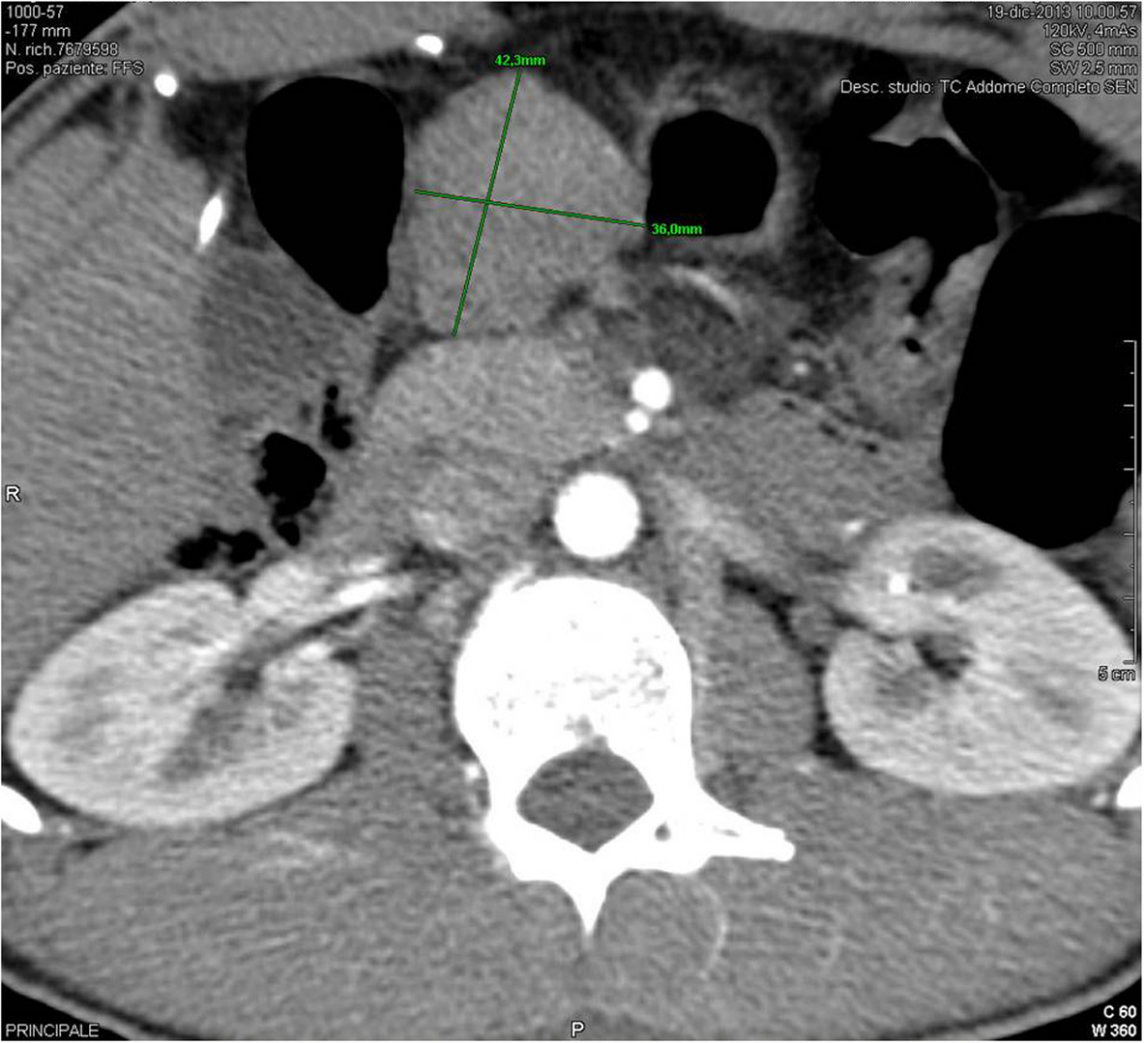
**Abdominal CT-scan with contrast: solid round lesion (42×36cm) adjacent to the left liver lobe, the stomach and the pancreas.**

An abdominal computed tomography (CT-scan) with contrast was executed to characterize the lesion. It shown a large solid round lesion (42×36mm) in the context of the mesenteric adipose tissue, under the left liver lobe, in contact medially with the lesser curvature of the stomach and behind with the pancreas (Figure 1). The lesion shown a faint and inhomogeneous enhancement in the arterial phase, which persisted in the venous phase. Other abnormalities to lymph nodes and the abdominal organs were not visualized. The neck and thoracic CT-scan did not reveal the presence of other lesions or masses, except for the known skeletal dysmorphia. The upper gastrointestinal contrast study did not visualized any external compression of the stomach.

**Figure 2:**
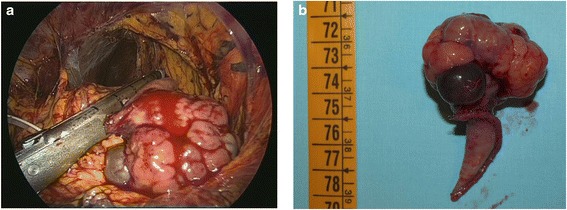
**Intraoperative finding. a**: the solid nodular mass was cut with 10mm Stappler. **b**: surgical specimen measuring 4 cm in diameter.

Laparoscopic surgical approach was conducted to evaluate the extension of the lesion and to make diagnosis of the origin of the mass. A subombelical incision and the opening of the peritoneum was realized to insert the 5 mm trocar. The CO2 was insuffled with a flow of 1 L/min and an intra-abdominal pressure of 10 mmHg. The 0° optic was inserted and two other trocars, 5 and a 10 mm, were positioned in the right and the left flank respectively. The exploration of the abdominal cavity was difficult for the presence of several omental adhesions, partially removed with a dissector and the LigaSure. The ventriculoperitoneal shunt was visualized in the right hypochondrium. At the posterior margin of the body of the stomach a 4 cm solid and nodular formation was visualized and handled with endoscopic blunt tip dissectors. A 10 mm Stappler was positioned in a macroscopically tumor-free area of the stomach and the lesion was cut. The specimen was finally inserted in an Endobag and removed through the umbilical wound (Figure 2a,b). A sample of omentum was removed with another endobag for the histopathologic examination. After the control of the hemostasis, the trocars were removed and the abdominal wall sutured. The patient tolerate the procedure well and his postoperative course was unremarkable.

**Figure 3:**
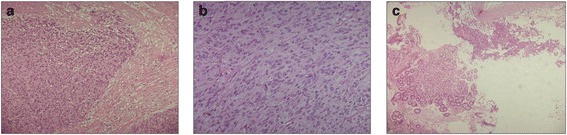
**Histopathologic examination of the mass: spindle and epithelioid cells, low mitotic rate (less than 5 × 50 HPF), no evidence of necrosis, erosions of the mucosa and serous. a**: GIST’s cells and gastric wall’s cells 10 ×. **b**: GIST’s cells 20×. **c**: GIST’s cells and gastric wall’s cells.

**Figure 4:**
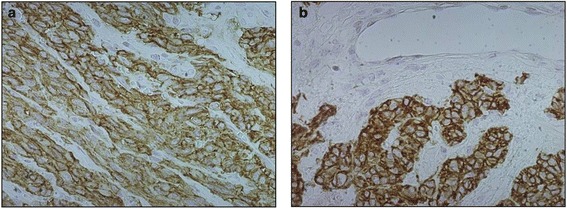
**Immunohistochemical examination. a**: tumor cells stained positive for CD117-c-KIT. **b**: positive for DOG-1.

Histopathologic examination of the mass revealed the presence of epithelioid and spindle cells with low mitotic activity (less than 5 × 50 high-power field (HPF)), erosions of the mucosa and serous and no evidence of necrosis (Figure 3a,b,c). Surgical margins of the mass and the omentum were microscopically free of tumor cells. Tumor cells stained positive for CD117 (Figure 4a) and DOG-1 (Figure 4b), negative for S-100, desmine, vimentine, smooth muscle actin, and neuron-specific enolase by immunohistochemistry. Genetic analysis were performed searching CKIT mutations (exons 9, 11, 13, 14, 17) and PDGFRA mutations (exons 12,14,18). In both genes, mutation were absent. So the tumor was characterized as a kit-wilde type GIST.

These results justify the decision not to add other surgical or medical therapy. However, for high risk of recurrence and metastasis, a close follow-up will be conducted.

## Discussion and evaluation

Gastrointestinal stromal tumors (GIST) are the most common mesenchymal tumors of the gastrointestinal tract (Gunaydin et al. 2012; Benesch et al. 2009; Durham et al. 2004; Guhyun K et al. 2013; Park et al. 2006; Jagannathan et al. 2012; Hayashi et al. 2005) accounting for 6.5 to 14.5 per million per year (Benesch et al. 2009). Men older than 40 years are predominantly affected (95% of all) (Gunaydin et al. 2012; Park et al. 2006; Jagannathan et al. 2012), with a peak incidence in the sixth and seventh decades (Gunaydin et al. 2012; Guhyun K et al. 2013; Park et al. 2006; Hayashi et al. 2005; Tran et al. 2013). Among children and adolescents GIST are very uncommon (Benesch et al. 2009; Hayashi et al. 2005) and have two peaks of incidence: less than 1-year-old and between 10 and 15 years old (Hayashi et al. 2005). The true incidence of pediatric GIST is not available for the rarity of the pathology and the lack of a prospective standardized registration (Benesch et al. 2009). Furthermore, the immunohistochemistry has recently allowed the accurate differentiation of GIST from other mesenchimal tumors such as leiomyomas, leiomyosarcomas, and plexosarcomas (Durham et al. 2004). The most accurate epidemiological data on paediatric GIST come from the UK National Registry of Childhood Tumours showing an annual incidence of 0.02 per million children below the age of 14 years (Benesch et al. 2009; Guhyun K et al. 2013) representing 1.4% of all GIST cases (Park et al. 2006; Jagannathan et al. 2012; Tran et al. 2013).

GIST can occur sporadically or concurrently with genetic syndromes, including familial GIST with germline KIT mutation, neurofibromatosis type 1, Carney triad and Carney–Stratakis syndrome (Benesch et al. 2009; Guhyun K et al. 2013; Park et al. 2006; Vaughan et al. 2011). Carney triad, firstly described in 1977 by J. Aidan Carney, associates gastric leiomyosarcoma, extra-adrenal paraganglioma and pulmonary chondroma (Benesch et al. 2009; Park et al. 2006; Vaughan et al. 2011). Carney–Stratakis syndrome or Carney–Stratakis is an inherited tumor syndrome comprising GIST and paragangliomas (Benesch et al. 2009; Vaughan et al. 2011).

GIST derived from the interstitial cells of Cajal (ICC) (Tran et al. 2013) that are distributed along the gastrointestinal tract and are considered the pacemaker for muscular motility of the gastrointestinal tract (Durham et al. 2004). Approximately 60% of GIST in adults presents the mutations of c-*kit* in the juxtamembranous domain. The alteration of this proto-oncogene leads to a “gain of function” promoting growth or preventing apoptosis (Durham et al. 2004). In up to 85% of paediatric cases, KIT or PDGFRA (*Platelet-derived Growth Factor Receptor Alpha*) mutation is absent (non-mutation kit genotype, termed kit-wilde type (WT GIST). However, the expression of phosphorylated KIT or the biochemical activation of KIT downstream molecules (AKT, mTOR, PDK1, MAPK) in the WT-GIST, results, similarly, in the uncontrollable proliferation of ICC. The lack of KIT gene mutation may result in decreased efficacy in target-based therapy with tyrosine kinase receptor inhibitors (Benesch et al. 2009; Tran et al. 2013).

These tumors can arise anywhere in the GI tract, but the vast majority, both in adults and in children, occurs in the stomach (50–60%) (Benesch et al. 2009; Guhyun K et al. 2013; Park et al. 2006; Hayashi et al. 2005; Tran et al. 2013) and in the small intestines (20–30%) (Gunaydin et al. 2012; Durham et al. 2004; Hayashi et al. 2005; Tran et al. 2013). Occurrence of multiple tumors is observed both in syndromic and sporadic form and regional recurrence rates are high in pediatric gastric GIST (Park et al. 2006). The liver (19%), the peritoneum (16%) and lymph nodes (11%) are the most common sites of metastasis; more rarely the lungs, pleura, bones, subcutaneous tissues (Jagannathan et al. 2012) and brain (one case reported) (Benesch et al. 2009; Jagannathan et al. 2012).

GIST are asymptomatic in 10% to 30% of patients (Gunaydin et al. 2012; Benesch et al. 2009) or present nonspecific symptoms and signs (Benesch et al. 2009; Tran et al. 2013). In many cases they are discovered incidentally during imaging, endoscopy or laparotomy for unrelated problems (Gunaydin et al. 2012). The most common clinical features are anaemia (39%), abdominal pain (16%), palpable abdominal mass (10%), gastrointestinal acute or chronic bleeding with hematemesis or melena, anaemia related symptoms, fatigue, dizziness, exertional dyspnea, or syncope (Gunaydin et al. 2012; Durham et al. 2004; Park et al. 2006; Tran et al. 2013). Rarely the mass could cause partial intestinal or biliary obstruction, dysphagia, hemoperitoneum, intussusception (Gunaydin et al. 2012; Park et al. 2006; Tran et al. 2013).

The differential diagnoses for large left upper quadrant mass include embryonal sarcoma, cystic mesenchymal hamartoma, primary pancreatic tumor, splenic pseudocyst, adrenal cortical carcinoma and primitive neuroectodermal tumor (PNET)/Askin tumor arising from the left hemidiaphram (Park et al. 2006).

These tumors present usually irregular, lobulated and ulcerated (Durham et al. 2004). They involve the muscularis propria with extramural, mural, and intramural extension (Tran et al. 2013; Park et al. 2006; Durham et al. 2004) or can be exophytic with a small pedicle (Park et al. 2006).

CT-scan of the abdomen and pelvis or magnetic resonance imaging (MRI) are mandatory in the diagnostic work-up and in monitoring the disease, as well as in the detachment of hepatic metastases (Benesch et al. 2009; Durham et al. 2004; Tran et al. 2013). CT-scan shows single or multiple solid-nodular masses which does not enhance with intravenous contrast (Durham et al. 2004; Park et al. 2006; Tran et al. 2013). MRI shows a rounded variable T1 and T2 signal with areas of necrosis and hemorrhage, that does not significantly enhance with intravenous gadolinium (Durham et al. 2004; Park et al. 2006; Tran et al. 2013). The lesions correspond to well circumscribed, often round filling defects on gastrointestinal contrast series that were absent in the case presented. In this case we have been obliged to choose the CT scan because of the presence of a cochlear implant that contraindicated MRI. Since GIST has the propensity for submucosal growth, additional endosonography can be utilized (Benesch et al. 2009). Fluorodeoxyglucose-positron emission tomography (FDG-PET) is useful for the diagnosis, the monitoring of the disease progression and treatment response (Gunaydin et al. 2012; Benesch et al. 2009; Tran et al. 2013) and the evaluation of metastases (Durham et al. 2004; Tran et al. 2013).

The final diagnosis is based on histology and immunohistochemistry. Tissue samples can be obtained either by (endoscopic or percutaneous) biopsy or resection of the tumor. Whereas endoscopic biopsies are often non-diagnostic and percutaneous biopsies may increase the risk of hemorrage and intra-peritoneal tumor spillage, surgical tumor resection is preferred (Gunaydin et al. 2012; Bhatnagar 2010). The mass, on preoperative imaging, has the characteristic of a sarcoma. Therefore, considering the presence of a single lesion, the surgical approach was decided to get a clear diagnosis, without further investigations. Intraoperatively, the mass was removable, so we performed a complete resection. The histopathologic examination of a GIST usually reveales spindle cells with atypical nuclei, organized in perpendicular bunches (Gunaydin et al. 2012; Durham et al. 2004). On immunohistochemistry assay the tumor cells stained uniformly for stem cell factor CD34 and c-kit (CD-117), variably positive for SMA and S-100, and negative for desmin (Gunaydin et al. 2012; Durham et al. 2004). Molecular biological examination detects KIT or PDGFRA mutations in 78% of GIST in young adults and PDGFRA mutants seem to be more frequent in younger patients (17% vs 4%). Recently, germline mutations encoding the succinate dehydrogenase (SDH) subunits (SDH B, SDH C, and SDH D) have been identified in Carney triad-associated and pediatric GIST (Guhyun K et al. 2013; Jagannathan et al. 2012). Thus it has been proposed that GIST can be divided into two distinctive subgroups on the basis of SDHB IHC. Type 1 (SDHB positive) tumors are common in adults, can occur anywhere in the gastrointestinal tract, have an equal gender distribution, and usually have KIT or PDGFRA mutations. Type 2 (SDHB negative), are typical in childhood and young adulthood and occur almost exclusively in the stomach (Guhyun K et al. 2013). Another mutation that can be founded in these tumors is BRAF mutation (in 1% of cases). There are no data about the significance of these mutation in term of prognosis.

Surgery is the first-line treatment (Gunaydin et al. 2012; Benesch et al. 2009; Hayashi et al. 2005) but there are no consensus guidelines and no prospective therapeutic treatment studies (Hayashi et al. 2005): current guideless for management and treatment of pediatric GISTs are based on clinical experience and data from case reports and case series (Tran et al. 2013).

For non metastatic resectable tumors, complete surgical resection is the gold standard.

Gross surgical resection with negative microscopic margins may lead to full remission (Gunaydin et al. 2012; Benesch et al. 2009; Durham et al. 2004; Hayashi et al. 2005; Tran et al. 2013) and endoscopic approach could be preferred in the treatment of small-sized GIST (Gunaydin et al. 2012). Resection of localized GIST of the stomach is allowed by antrectomy, partial (distal) gastrectomy or by wedge resection, subtotal or total gastrectomy (Benesch et al. 2009). We decided for a laparoscopic wedge resection and an omentectomy to exclude its involvement. Currently, laparoscopy is gaining an increasingly important role in oncology, especially for small lesions and in cases in which oncologic criteria can be respected with this technique. Stating that preoperative imaging didn’t revealed lymph-node involvement, we didn’t performed lymphadenectomy waiting for a correct diagnosis from histology. Futhermore, intraoperatively we noted several adhesions due to previous surgery that made difficult to reach lymphatic stations.

In several case series, 20-29% of pediatric patients have metastases at diagnosis, usually localized in the liver, lymphnodes, and peritoneum (Janeway and Weldon 2012). Since lymph node involvement is greater in pediatric patient, an active search for occult lymph node involvement sould be performed when a GIST is suspected and the procedure is technically possible. Nevertheless, in case of diffuse disease, extensive lymphadenectomy has not been shown to improve survival (Durham et al. 2004; Hayashi et al. 2005).

During surgical procedure it is important to avoid tumor rupture/spill as this is associated to high risk of recurrence. In Fact some Authors reported a recurrence rate of 78% in patients with intraoperative or preoperative tumor rupture (Janeway and Weldon 2012).

The use of conventional cytotoxic chemotherapy is not recommended for the treatment of pediatric GIST because they give poor responses (Benesch et al. 2009; Durham et al. 2004; Hayashi et al. 2005; Bhatnagar 2010). Currently, an oral protein tyrosine kinase inhibitor, imatinib mesylate, has shown promising antiproliferative and proapoptotic effects against the mutated, constitutively active transmembrane CD-117 receptor found in most GIST (Durham et al. 2004). After the approval by Food and Drug Administration (USA) in 2002, this drug found increasing application for adult GIST in adjuvant therapy, improving the overall tumor-free survival and reducing of recurrences (Bhatnagar 2010). Thus, all tumors should be evaluated for KIT and PDGFRA mutations to determine efficacy of targeted therapy with imatinib or sunitinib (Benesch et al. 2009). Sunitimib is a tyrosine-kinase inhibitor 10 times more potent than imatinib that seems to be effective in adult patients with imatinib-resistant GIST, but there are no sufficient data to establish the real efficacy in pediatric patients (Janeway KA & Pappo 2012).

WT GIST had poor or absent response to TKI and the adjuvant therapy with these drugs seems not to increase the recurrence free survival. Moreover, emerging data suggest that long-term exposure to imatinib could have toxic effect on pediatric patient, so the use of TKI isn’t presently recommended.

Paediatric GIST have a relatively benign clinical course despite a high rate of metastases, tumor recurrence and limited response to tyrosine kinase inhibitors (Gunaydin et al. 2012; Benesch et al. 2009; Jagannathan et al. 2012; Tran et al. 2013). Recurrence after complete remission involves 25% of patients in most series, but less than 10% of patients with metastatic disease die because of GIST. The 5-year survival rates for GIST ranges from 32% to 63% after complete surgical resection (Durham et al. 2004).

Few risk stratification schemes have been demonstrated to predict risk of recurrence in adults. Principal criteria are tumor size, tumor site, mitotic index, tumor rupture. According to these criteria, primary tumor size greater than 5 cm (Gunaydin et al. 2012; Durham et al. 2004), mitotic index greater than 5 mitoses per 50 HPFs, spindle cell morphology, shorter disease-free interval, and non-resected metastatic disease are associated with a worse prognosis (Gunaydin et al. 2012). Unfortunately, the risk stratification criteria are probably not relevant to children with WT GIST.

Metastatic, unresectable WT GIST has a more indolent course and the mean survival from the development of metastases is 15 years. Considering the limits of medical therapy, cure is unlikely, so the goal should be the disease control and symptom management. Surgery should be considered because of the long time these patients can live with GIST, but aggressive procedure leaving macroscopic disease should be avoided and a proper counselling is mandatory considering that a complication rate of 60% is reported. In metastatic, unresectable WT GIST, a combined approach with TKI and surgery can be considered. Imatinib has some activity in adult with WT GIST but responses in children appear to be uncommon. Sunitinib appears to be more effective in pediatric and adult GIST but the effect is rare and is typically a disease stabilization. Other TKI are nilotinib and masitinib but their activity in WT GIST has not been assessed. Recently, insulin-like growth factor 1 receptor has been founded to be overexpressed in WT GIST and could be a new target for therapy.

Because of the high recurrence rate, a strict surveillance is recommended. According to most of Authors, axial imaging with CT scan every 3–6 month for the first 5 years after surgery represent a useful tool for the follow up. MRI could be used instead of CT to avoid excessive radiation exposure. In this case, MRI was contraindicated because of the presence of cochlear implant, so we decided to use abdominal ultrasounds and chest X-Ray alternatively to CT scans every 4–6 months. Currently, the disease is in remission at 11 months after surgery.

## Conclusions

It is widely accepted that surgery is the first-line treatment in children and adolescents with resectable GIST (Gunaydin et al. 2012; Benesch et al. 2009; Hayashi et al. 2005) and gross resection with negative microscopic margins may lead to full remission (Gunaydin et al. 2012; Benesch et al. 2009; Durham et al. 2004; Hayashi et al. 2005; Tran et al. 2013). Laparoscopy could be considered a safe and effective surgical approach for the exploration of the abdominal cavity to evaluate the extension of the disease and for the complete removal of the tumor.

## Consent

Written informed consent was obtained from the patient's parent for the publication of this report and any accompanying images.

## Abbreviations

GIST: Gastro-intestinal stromal tumor
GI: Gastro-intestinal
PDGRFA: Plateled-derived growth factor receptor alpha
HPF: High power field
ICC: Interstitial cells of Cajal
WT: Wild type
MRI: Magnetic resonance imaging
CT: Computed tomography
FDG-PET: Fluorodeoxyglucose positrone emission tomography
SDH: Succinate dehydrogenase
TKI: Tyrosine kinase inhibitor
